# Clinical and Arthroscopic Findings of Acute Anterior Cruciate Ligament Tears of the Knee

**DOI:** 10.1155/DTE.2.107

**Published:** 1995

**Authors:** Kenji Shirakura, Masanori Terauchi, Naoki Fukasawa, Masashi Kimura, Takachika Shimizu

**Affiliations:** 1 Department of Orthopaedic Surgery Gunma University School of Medicine 3-39-22 Showa-machi Gunma-ken Maebashi-shi 371 Japan; 2 Gunma Sports Medicine Research Center Zenshukai Hospital Gunma-ken Japan; 3 Geriatrics Research Institute and Hospital Gunma-ken Japan

## Abstract

Clinical, arthrographic, and arthroscopic findings in 53 patients with acutely torn anterior cruciate ligaments (ACLs) were documented. Arthroscopy and instability tests under anesthesia were performed on all patients within 2 weeks after the initial injury. Twenty-three patients complained of extension blocks, and localized tenderness on the medial side was revealed in 26 patients at the initial examination. Aspiration from joints exhibited hemarthrosis in 52 patients. Arthroscopy revealed ACL ruptures in all patients. Four Segond's fractures, 26 meniscus tears (8 medial and 18 lateral), 1 osteochondral fracture, and 19 medial collateral ligament ruptures were revealed. Arthroscopy detected only 1 of the 5 ruptures of the posteromedial corner of the medial meniscus, which were noted on arthrography. Three ACL stumps were protruding among the femorotibial joint, which seemed to be restricting full extension. Statistical analysis showed that tenderness on the medial side was not revealed more frequently in knees with medial collateral ligament injuries than in the others. The volume of aspirated fluids in knees with no leakage in arthrography significantly increased over those with leakages (*p* < 0.05). Diagnosis of ACL injuries should be completed by clinical, arthrographic, and arthroscopic examinations.


*Diagnostic and Therapeutic Endoscopy,* 1995, Vol. 2, pp. 107–112
Reprints available directly from the publisher
Photocopying permitted by license only

© 1995 Harwood Academic Publishers GmbH
Printed in Singapore

### Clinical and Arthroscopic Findings of Acute Anterior Cruciate Ligament Tears of the Knee

KENJI SHIRAKURA, MASANORI TERAUCHI, NAOKI FUKASAWA, MASASHI KIMURA and TAKACHIKA SHIMIZU

*Department of Orthopaedic Surgery, Gunma University School of Medicine (K.S., M.T., N.K.), Gunma Sports Medicine Research Center, Zenshukai Hospital (M.K.), and Geriatrics Research Institute and Hospital (T.S.), Gunma-ken, Japan*

*(Received March 14, 1995, in final form, May 18, 1995)*

Clinical, arthrographic, and arthroscopic findings in 53 patients with acutely torn anterior cruciate ligaments (ACLs) were documented. Arthroscopy and instability tests under anesthesia were performed on all patients within 2 weeks after the initial injury. Twenty-three patients complained of extension blocks, and localized tenderness on the medial side was revealed in 26 patients at the initial examination. Aspiration from joints exhibited hemarthrosis in 52 patients. Arthroscopy revealed ACL ruptures in all patients. Four Segond's fractures, 26 meniscus tears (8 medial and 18 lateral), 1 osteochondral fracture, and 19 medial collateral ligament ruptures were revealed. Arthroscopy detected only 1 of the 5 ruptures of the posteromedial corner of the medial meniscus, which were noted on arthrography. Three ACL stumps were protruding among the femorotibial joint, which seemed to be restricting full extension. Statistical analysis showed that tenderness on the medial side was not revealed more frequently in knees with medial collateral ligament injuries than in the others. The volume of aspirated fluids in knees with no leakage in arthrography significantly increased over those with leakages ($p < 0.05$). Diagnosis of ACL injuries should be completed by clinical, arthrographic, and arthroscopic examinations.

**KEY WORDS:** Anterior cruciate ligament injury, arthrography, associated injury, diagnostic arthroscopy, locking

### INTRODUCTION

The diagnosis of acute anterior cruciate ligament (ACL) tears has been secured because of the characteristic mechanisms of injury and the concomitant hemarthrosis (1). The Lachman test is a reliable means of demonstrating acute ACL involvement, whereas a tense hemarthrosis and the protective spasms of the hamstring muscles preclude the knee motion (2). Clinical evaluation, however, for the status of ACL in the face of injury, must include a consideration of the status of all other joint structures (3). Several reports (1,3–11) of patients with ACL injuries examined by clinical and arthroscopic means have been published, but there are no reports in which the same clinician carried out the initial clinical and arthroscopic examination during the acute phase. The authors performed a prospective study to document the details of clinical, arthrographic, and arthroscopic findings of acute ACL injured knees.

Address for correspondence: K. Shirakura M.D., Ph.D., Department of Orthopaedic Surgery, Gunma University School of Medicine, 3-39-22 Showa-machi, Maebashi-shi, Gunma-ken, Japan 371.

### MATERIALS AND METHODS

The subjects were 53 consecutive patients on whom arthroscopy was performed between April 1983 and May 1991 by one of the authors (K.S.). The following criteria were used for the patient selection: 1) patients who had sustained acute ACL tears that were not associated with posterior cruciate ligament tears; 2) patients in whom the ACL was injured midway between the ends of the ligament and had no bony fragments at the end of the stump; 3) patients in whom arthroscopy was performed within 14 days after the time of the initial injury; and 4) patients on whom the primary ACL repair and/or reconstruction was not performed.

The subjects included 23 men and 30 women with injuries to 23 right knees and 30 left knees. Ages ranged from 14 to 60 years with a mean of 29.4 ± 11.1 years. Forty-six injuries occurred during sports activities, four during traffic accidents, and three during industrial accidents. Injuries in a fall during skiing were considered to be contact injuries. Twenty-four were involved in noncontact injuries. Arthroscopy and concomitant procedures in the acute phase were performed as early 2 days and as late as 14 days postinjury, with a mean of 7.4 ± 3.4 days.

Physical and roentogenographic examinations were performed and recorded at the patients' initial visit to the hospital after their injuries. Examinations consisted of volume measurements and color inspections of the joint fluid, measurements of the range of active motion, and location of tenderness to palpating. The routine roentogenographic features were provided by anteroposterior, lateral, and skyline views. Arthrography was performed on 43 of the 53 patients before arthroscopy. All patients had received arthroscopy and instability tests under anesthesia. Arthroscopy was completed through the lateral infrapatellar approach with a 5.0 and/or 4.0 mm right angle Shinko scope (Tokyo, Japan). The probes were used routinely to facilitate intra-articular inspection through the second portal.

ACL tears were divided into two types, the complete type and the residual type, arthroscopically. Those knees with residual tears demonstrated definite instabilities, but some tissue remained between the ACL origin and the insertion.

Medial collateral ligament (MCL) tears were classified according to Fetto and Marshal's criteria (12). Intra-articular procedures through the second portal were performed during arthroscopic examinations. The MCL tears for which the primary sutures were supposed to produce a satisfactory restration were repaired simultaneously.

The $t$ test and $\chi^2$ test were used for the statistical analysis.

### RESULTS

### Clinical Findings

All but one patient showed a restricted range of motion of the knee joint in the acute phase. Twenty-three patients complained of a painful extension block, ranging from 5 to 30° with an average of 13.3 ± 7.6°. Since 52 patients could not flex their knees completely, the flexion angles were limited to a minimum of 20° and a maximum of 140° with an average of 86.0° ± 30.2°. Three patients exhibited fixed knees at angles of 20° to 30° of flexion, and they could not extend nor flex their knees any further. Aspiration from the joint exhibited hemarthrosis in 52 patients, and the volume of the fluid ranged from 5 to 80 ml with mean of 28.5 ± 19.8 ml (Fig. 1). Localized tenderness was readily revealed in a total of 36 areas in 32 patients, 26 of which were located in the medial, and 6 of which were in the lateral side of the joint.

### Radiographic Findings

Radiographs revealed Segond's fractures in 4 knees, and this included all of those that showed a bony involvement. Arthrography detected 2 lateral and 5 medial meniscus tears, as well as 12 leakage of the contrast medium from the joint capsule in 11 patients. Medial meniscus ruptures were all longitudinal tears involving the posteromedial segment of the medial meniscus. Leakages of contrast medium were noted in 10 medial, 1 lateral, and 1 posterolateral areas of the 12 knees, respectively. The volumes of aspirated fluids in knees with leakages ranged from 5 to 30 ml (mean, 15.9 ± 7.7 ml), and those with no leakage ranged from 5 to 80 ml (mean, 27.9 ± 18.6ml). A significant difference was noted ($t$ test, $P < 0.05$).

### Instability Tests

Results of instability tests under anesthesia are shown in Figure 2. The Lachman test and pivot shift sign revealed much higher sensitivities than the anterior drawer tests (Fig. 2).

### Arthroscopic Findings

Arthroscopy detected ACL ruptures in all the 53 patients. Fifteen ACLs were revealed to be ruptures of the residual type (Fig. 3). Thirty-eight ACLs were revealed to be complete ruptures. Fourteen of the 15 with residual type ruptures revealed positive Lachman test results and a pivot shift sign. One of the 15 patients showed a positive pivot shift sign, but negative Lachman test results. Of 38 pa-

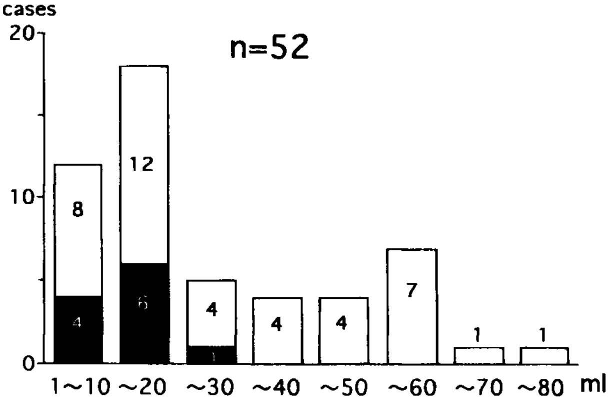

**Figure 1** Distributions of the volumes of aspirated joint fluid. ■, patients with leakages seen on arthrography.

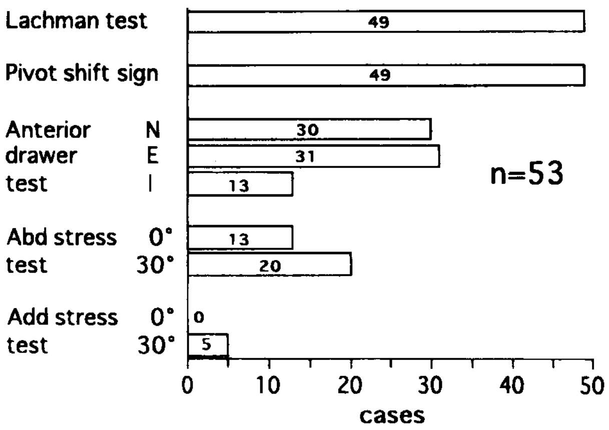

**Figure 2** Number of patients with positive instability tests under anesthesia. N, with tibia in the neutral position; E, with tibia externally rotated; I, with tibia internally rotated; Abd, abduction; Add, adduction.

tients with complete ACL tears, 2 patients showed negative Lachman test results and a negative pivot shift sign. One patient showed negative Lachman test results, and 2 patients showed negative pivot shift signs. In those patients the diagnosis was based on arthroscopic findings. The frequencies of positive instability test results revealed no significant difference between the complete and residual type ACL tears ($\chi^2$ test).

Arthroscopy revealed 18 lateral meniscus tears. Sixteen of these had small longitudinal tears ranging from 2 to 10 mm, which had not torn completely through the full thickness of the posterior segment. The other 2 lateral meniscus injuries were complex type ruptures of the posterior segment, which were noted on arthrography. Three medial meniscus tears, other than the 5 noted on arthrography, were also detected arthroscopically. In total, 18 lateral meniscus injuries, 8 medial meniscus injuries (3 bilaterally involved), 1 osteochondral fragment detached from the medial femoral condyle, and 19 MCL ruptures were revealed in the acute phase. Among the 19 MCL ruptures, 1 was of Grade 1 severity, 4 were of Grade 2, and 14 were of Grade 3.

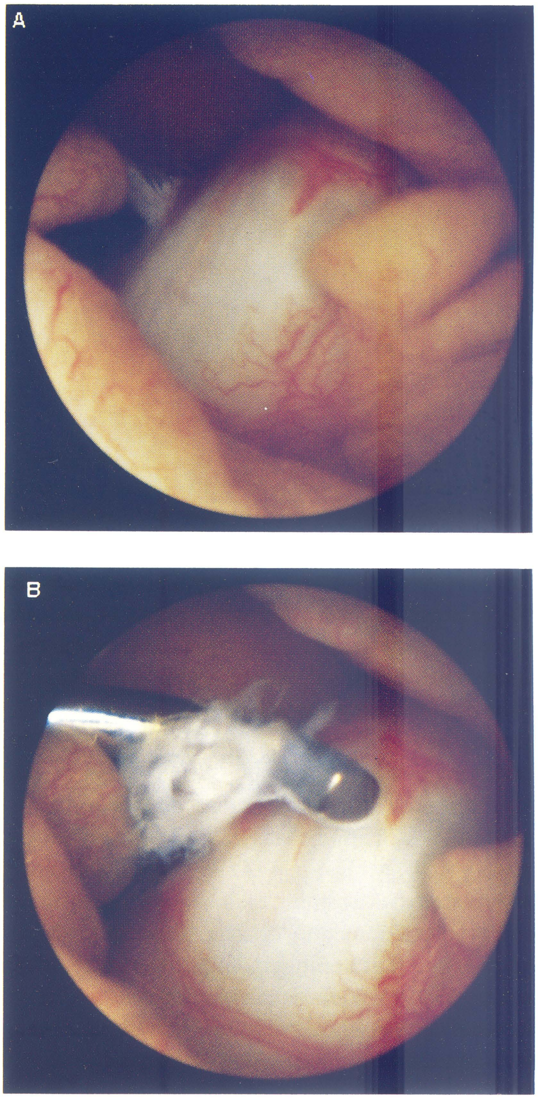

**Figure 3** Arthroscopy of an ACL tear of residual type (left knee). A. The ruptured ACL fibers were exposed on the synovial sheath. B. The fibers came to be clearly visualized by probing.

### Procedures

As probing did not demonstrate any abnormal mobility of the injured segments, the 18 lateral and 7 medial meniscus tears did not require surgical treatment. One ACL stump was protruding among the femorotibial joint, which seemed to be restricting full extension. One medial meniscectomy, 1 removal of an osteochondral fragment, and 1 excision of the ACL stump were performed under arthroscopy. Primary ligament sutures were performed on 10 of the 19 MCL injuries immediately after arthroscopy.

Two patients exhibited a repeat giving-way phenomenon, and the restriction of a full extension in the subacute phase continued. Repeat arthroscopy documented the fact that rounded ACL stumps interposed between the femur and tibia were disturbing the extension (Fig. 4). These stumps were excised under arthroscopy at the 3rd and 8th week after the time of first arthroscopy, respectively.

As a result, in 13 of 19 patients with MCL injuries a tenderness on the medial side of the knee was revealed at the initial examination. Thirteen of 34 with no MCL injury had tenderness on the medial side. Statistical analysis using the $\chi^2$ test showed that tenderness on the medial side was not revealed more frequently in the knees with MCL ruptures than in the knees without MCL injuries.

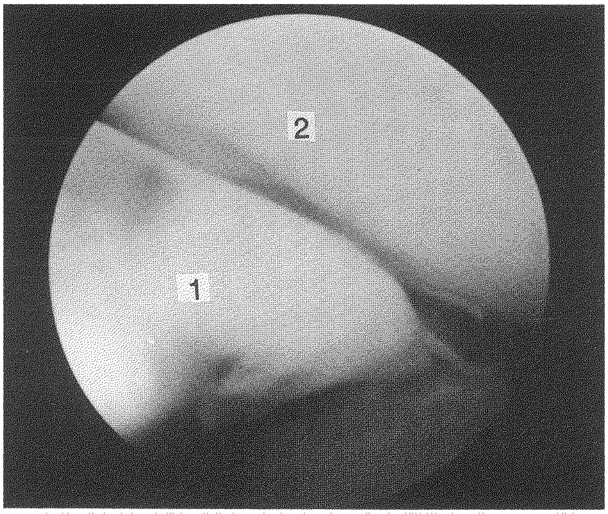

**Figure 4** ACL stump among the medial femorotibial joint. Repeat arthroscopy revealed a protruding ACL stump among the medial femorotibial joint at the 3rd week after the initial arthroscopy. Excision of the stump allowed the knee to.extend fully. 1, ACL stump; 2, medial femoral condyle.

### DISCUSSION

McMaster et al (13). suggested that the block in the joint with an acute ACL injury was caused by the entrapment of the torn ACL. McDaniel (14), however, believing that locking related to ACL injuries does not truly exist, terms the observed phenomenon "pseudo-locking." Monaco et al (15). inferred that impingement was visualized directly in one-third of the locked knees with acute ACL tears, while the other two-thirds regained full extension under anesthesia. Allum and Jones (16) investigated 50 locked knees with a variety of conditions, and pointed out that the mean loss of extension was 24° and that 68% were tender over the medial joint line. Noyes et al. (6) detected the tenderness to palpation medially in 84% of patients with acute traumatic hemarthrosis of the knee. The extension block and predominant tenderness on the medial side are common features of acute hemarthrosis and are not indicative of associated injuries. Locking was not always caused by a true mechanical block; in some patients a torn ACL disturbed the full extension. The long ACL stub should be excised or reduced under arthroscopy when it protrudes into the joint and the ACL has no opportunity to heal.

There were no overt findings, except for the Segond's fractures on the plain radiographs in the present study. Early investigators of acute hemarthrosis did not refer to this fracture. The incidence of Segond's fractures in patients with ACL injuries in this study was 8%, a figure that was not significantly different from those of other investigators (11). This fracture represents an avulsion injury to the inferior meniscal capsular attachment of the lateral meniscus (17). Woods termed this sign the "lateral capsular sign," and emphasized that injured knees with this fracture were supposed to have sustained combined ACL and MCL injuries (18). In support of this opinion, three of the four patients with this fracture in this study had an ACL tear associated with an MCL tear.

**Table 1** Detection of Meniscus Injury

| | Arthrography | Arthroscopy | Both | Total |
|---|---|---|---|---|
| Medial | 4 | 3 | 1 | 8 |
| Lateral | 0 | 16 | 2 | 18 |

Kennedy et al. (19) implied that the arthroscopic appearance of the ACL did not provide an accurate representation of its functional capacity. In patients with ACL tears of the residual type, the authors of the present study relied upon instability tests under anesthesia. Some authors have reported relatively good results with treatment of partial ACL ruptures as opposed to complete tears (20–22). Those authors described the criteria of "incomplete tear" as being an ACL that had partial tears but showed no sagittal instability. The frequencies of positive instability test results under anesthesia revealed no significant difference between the complete and residual type ACL tears. ACL tears with some remaining tissue under arthroscopy in the present study did not represent a partial rupture of the ACL fibers.

Many authors have reported a surprisingly high incidence of associated tears of the meniscus with an acutely torn ACL (1,3,4,8,11). They emphasized that the majority involved the posterior horn of the lateral meniscus. Indelicato et al. (8), however, reported a higher incidence of peripheral posterior horn tears of the medial meniscus. They used the second portal, the posteromedial approach, which may cause damage to the saphenous nerve (23,24). In the present study, arthroscopy detected only one of the five ruptures of the posteromedial corner of the medial meniscus, which were all clearly noted on the arthrogram in the acute phase (Table 1).

Kimori et al. (25) indicated the difficulties of evaluating injuries of the posteromedial corner by arthroscopy alone and/or by other imaging techniques and emphasized the necessity for arthrography. Gershuni et al. (26) concluded that the healing of meniscal tears is vastly improved when the knee is firmly immobilized and weight-bearing is prohibited. Meniscal involvement must be detected when the conservative treatment for an acutely torn ACL is under consideration. The posteromedial corner of the knee with an ACL injury should be carefully evaluated, not only by arthroscopy but by other means of diagnosis as well.

Nonoperative management has been widely accepted as the prefered treatment for an isolated MCL injury. Investigators who recommend the conservative method of treatment for MCL tears limit their recommendations to isolated MCL tears, but not to combined ACL/MCL injuries (27–30 ). Some authors have reported unfavorable results after conservative or operative treatment for MCL tears combined with ACL ruptures (5,12,31). Ballmer et al. (32) and Shelbourne et al. (33) recommended an isolated ACL reconstruction as the initial treatment for combined ACL/MCL injuries. Hughston et al. (34) thought that a repair of the medial ligaments should be performed on knees with these combined injuries. A quantitative evaluation of MCL injury is necessory and operative management for either an ACL or MCL should be considered when the knee sustains a severe MCL injury combined with an ACL rupture.

### CONCLUSIONS

The loss of extension and predominant tenderness on the medial side are common features of acute ACL injuries and are not indicative of associated injuries. Some extension blocks, however, are caused by the interposition of the ACL stump. The remaining tissue of a residular type ACL tear did not affect the results of the instability tests under anesthesia. Arthroscopy alone is not enough to evaluate injuries of the posteromedial corner of the knee. A quantitative evaluation of MCL injury is necessory, when the knee sustains an MCL injury combined with an ACL rupture. The diagnosis of ACL injuries should be completed by clinical, arthrographic, and arthroscopic examination.

### ACKNOWLEDGMENTS

This work was presented at the 16th Annual Meeting of the Japan Arthroscopy Association, Tokyo, Japan, 1990.

The authors wish to thank Professor Eiichi Udagawa and Kiichi Osawa for their contributions to the study.

### REFERENCES

1. Fetto JF, Marshall JL. The natural history and diagnosis of anterior cruciate ligament insufficiency. Clin Orthop 1980;147:29–38.
2. Torg JS, Conrad W, Kalen V. Clinical diagnosis of anterior cruciate ligament instability in the athlete. Am J Sports Med 1976;4:84–93.
3. Dehaven KE. Diagnosis of acute knee injuries with hemarthrosis. Am J Sports Med 1980;8:9–14.
4. Gillquist J, Hagberg G, Oretorp N. Arthroscopy in acute injuries of the knee joint. Acta Orthop Scand 1977;48:190–196.
5. Warren RF, Marshall JL. Injuries of anterior cruciate and medial collateral ligaments of the knee. Clin Orthop 1978;136:191–197.
6. Noyes FR, Bassett RW, Grood ES, et al. Arthroscopy in acute traumatic hemarthrosis of the knee: incidence of anterior cruciate tears and other injuries. J Bone J Surg 1980;62A:687–695.
7. Hawkins RJ, Misamore GW, Merritt TR. Follow-up of the acute non-operative isolated anterior cruciate ligament tear. Am J Sports Med 1986;14:205–210.
8. Indelicato PA, Bittar ES. A perspective of lesions associated with ACL insufficiency of the knee. Clin Orthop 1985;198:77–80.
9. Clancy WG, Ray M, Zoltan DJ. Acute tears of the anterior cruciate ligament. J Bone Jt Surg 1988;70A:1483–1488.
10. Shirakura K, Kato K, Udagawa E. Characteristics of the isokinetic performance of patients with injured cruciate ligaments. Am J Sports Med 1992;20:754–760.
11. Spindler KP, Schils JP, Bergfeld JA, et al. Prospective study of osseous, articular, and meniscal lesions in recent anterior cruciate ligament tears by magnetic resonance imaging and arthroscopy. Am J Sports Med 1993;21:551–557.
12. Fetto JF, Marshall JL. Medial collateral ligaments of the knee: a rationale for treatment. Clin Orthop 1978;132:206–218.
13. McMaster JH, Weinert CR, Scranton P. Diagnosis and management of isolated anterior cruciate ligament tears: a preliminary report on reconstruction with the gracilis tendon. J Trauma 1974;14:230–235.
14. McDaniel WJ Jr. Isolated partial tear of the anterior cruciate ligament. Clin Orthop 1976;115:209–212.
15. Monaco BR, Noble HB, Bachman DC. Incomplete tears of the anterior cruciate ligament and knee locking. JAMA 1982;247:1582–1584.
16. Allum RL, Jones JR. The locked knee. Injury 1986;17:256–258.
17. Pavlov H. The radiographic diagnosis of the anterior cruciate ligament deficient knee. Clin Orthop 1983;172:57–64.
18. Woods GW, Stanley RF, Tullos HS. Lateral capsular signs and X-ray due to a significant knee instability. Am J Sports Med 1979;7:27–33.
19. Kennedy JC, Hawkins RJ, Willis RB, et al. Tension studies of human knee ligament: yield point, ultimate failure, and disruption of the cruciate and tibial collateral ligament. J Bone Jt Surg 1976;58A:350–355.
20. Kannus P, Järvinen, M. Conservatively treated tears of the anterior cruciate ligament, long-term results. J Bone Jt Surg 1987;69A:1007–1012.
21. Odensten M, Hamberg P, Nordin M, et al. Surgical or conservative treatment of the acutely torn anterior cruciate ligament. Clin Orthop 1985;198:87–93.
22. Sandberg R, Balkfors B. Partial rupture of the anterior cruciate ligament: natural course. Clin Orthop 1987;220:176–178.
23. Stone RG, Miller GA. A technique of arthroscopic suture of torn menisci. Arthroscopy 1985;1:226–232.
24. Barber FA. Meniscus repair: results of arthroscopic technique. Arthroscopy 1987;3:25–30.
25. Kimori K, Suzu F, Yamashita F, et al. Evaluation of arthrography and arthroscopy for lesions of the posteromedial corner of the knee. Am J Sports Med 1989;17:638–643.
26. Gershuni DH, Skyhar MJ, Danzig LA, et al. Experimental models to promote healing of tears in the avascular segment of canine knee menisci. J Bone Jt Surg 1989;71A:1363–1370.
27. Ellsasser JC, Reynolds FC, Omohundro JR. The non-operative treatment of collateral ligament injuries of the knee in professional football players: an analysis of seventy-four injuries treated non-operatively and twenty-four injuries treated surgically. J Bone Jt Surg 1974;56A:1185–1190.
28. Hart DP, Dahners LE. Healing of the medial collateral ligament in rats: the effect of repair, motion, and secondary stabilizing ligament. J Bone Jt Surg 1987;69A:1194–1199.
29. Indelicato PA, Hermansdorfer J, Huegel P. Nonoperative management of complete tears of the medial collateral ligament of the knee in intercollegiate football players. Clin Orthop 1990;256:174–177.

30. Woo SL, Inoue M, McGurk-Burleson E, et al. Treatment of the medial collateral ligament injury. II. Structure and function of canine knees in response to differing treatment regimens. Am J Sports Med 1987;15:22–29.
31. Ciccoti MG, Lombardo SJ, Nonweiler B, et al. Non-operative treatment of ruptures of the anterior cruciate ligament in middle-aged patients: results after long-term follow-up. J Bone Jt Surg 1994;76A:1315–1321.
32. Ballmer PM, Ballmer FT, Jakob RP. Reconstruction of the anterior cruciate ligament alone in the treatment of a combined instability with complete rupture of the medial collateral ligament, a prospective study. Arch Orthop Trauma Surg 1991;110:139–141.
33. Shelbourne KD, Porter DA. Anterior cruciate ligament-medial collateral ligament injury: nonoperative management of medial collateral ligament tears with anterior cruciate ligament reconstruction. A preliminary report. Am J Sports Med 1992;20:283–286.
34. Hughston JC, Andrews JR, Cross MJ, et al. Classification of knee ligament instabilities. Part 1. The medial compartment and cruciate ligaments. J Bone Jt Surg 1976;58A:159–172.

